# Editorial: Multi-omics analysis in tumor microenvironment and tumor heterogeneity

**DOI:** 10.3389/fgene.2023.1271295

**Published:** 2023-08-23

**Authors:** Yuxin Shi, Qinglin Zhang, Jie Mei, Jinhui Liu

**Affiliations:** ^1^ Department of Oncology, The Affiliated Wuxi People’s Hospital of Nanjing Medical University, Wuxi People’s Hospital, Wuxi Medical Center, Nanjing Medical University, Wuxi, Jiangsu, China; ^2^ Department of Gastroenterology, The Affiliated Wuxi People’s Hospital of Nanjing Medical University, Wuxi People’s Hospital, Wuxi Medical Center, Nanjing Medical University, Wuxi, Jiangsu, China; ^3^ Department of Gynecology, The First Affiliated Hospital of Nanjing Medical University, Nanjing, Jiangsu, China

**Keywords:** multi-omics, cancer immunotherapy, tumor microenvironment, biomarker, tumor heterogeneity

Multi-omics technologies characterize biological activities by simultaneously integrating multiple single-modality omics methods, including the transcriptome, genome, epigenome, epi-transcriptome, proteome, metabolome, and developing omics (single-cell omics) (Jiang et al., 2022; Baysoy et al., 2023). A single-modality omics analysis to probe the cause of tumors and the direction of treatment has its limitations. Recent advances in multi-omics technology have proven to be the weapon of choice for dissecting the core mechanisms of cancer (Chai et al., 2021). As an encouraging case, the Cancer Genome Atlas (TCGA) serves to integrate multi-omics resources and has been successfully implemented in the analyzing of various cancer types (Liu et al., 2018; Sanchez-Vega et al., 2018). Multi-omics approaches also leave researchers with significant challenges. For instance, deficient necessitates standardization to allow sufficient integration across domains and appropriate methods for funneling complex information into clinical consequences (Hasin et al., 2017; Akhoundova and Rubin, 2022).

The tumor microenvironment (TME) is a complex system that contains cancer cells themselves, tumor-infiltrating immune cells, stromal cells, the extracellular matrix (ECM), and other secreted molecules (Lei et al., 2020). Dynamic interaction of all the components of TME contributes to malignant progression by generating heterogeneity, clonal evolution, and enhancing multidrug resistance in tumor cells (Tiwari et al., 2022). The coordination of several fundamental factors (such as innate immune-sensing machinery, oncogenetic alterations, metabolism alterations, and epigenetic regulators) can modulate anti-tumor responses by altering TME (Duan et al., 2020). Tumors could be roughly classified as cold or hot based on the features of the TME. Hot tumors are characterized by T cell infiltration and immune activation, presenting efficient response to immunotherapy, whereas cold tumors characterize oppositely. Therefore, reversing non-inflamed cold tumors into hot ones to achieve better immunotherapeutic response has emerged as a research focus (Duan et al., 2020; Mao et al., 2022; Mei et al., 2022; Mei et al., 2023).

Growing evidence suggests that TME is a dynamic network during tumor progression or therapeutic interventions (De Nola et al., 2021). Additionally, heterogeneity is present in almost all solid tumors and varies geographically and temporally with tumor progression and therapeutic intervention (Dentro et al., 2021). Anti-tumor immune heterogeneity is related to disease progression and therapeutic reactivity, particularly in immunotherapy (Wang et al., 2023). Thus, developing multi-omics profiling will enable in-depth characterization of diversified tumor types and better reveal their function in cancer immune escape.

It is an important research direction of precision medicine to systematically study clinical and pathological mechanisms and determine the best therapeutic targets through the multi-omics analysis of the TME. Subsistent multi-omics tools can reveal the heterogeneous composition of tumor and immune cells in the TME, to evaluate disease progression and guide the therapeutic regimens to each patient (Hsieh et al., 2022). Murai et al. (2023) performed multi-omics profiling to stratify nonviral hepatocellular carcinoma based on the TME features and identified the crosstalk between intratumoral steatosis and the TME. It benefits for identifying immunotherapy‐susceptible hepatocellular carcinoma, providing valuable insight into the development of precise therapy. Moreover, pyroptosis-related gene score was explored and combined with TME features in ovarian cancer patients to define three pyroptosis modification patterns and TME immune characteristics of these patterns, which can be used to develop accurate and personalized treatment strategies for ovarian cancer patients (Liu et al., 2022). In addition, multi-omics analysis can also accelerate the identification of biomarkers that predict efficacy and prognosis in multiple tumors. In the development of endometrial carcinoma, two immune-related genes were identified to construct the immune-related prognostic signature, which was closely associated with the infiltration of several types of immune cells and can predict the prognosis and therapeutic response of endometrial carcinoma patients (Liu et al., 2021).

Despite these research papers published in this Research Topic can provide unique insights into the application of multi-omics analysis in the TME and tumor heterogeneity, multi-omics cancer research still faces many challenges and difficulties. Making standardized panels for a certain entity and clinical context is a critical issue for many of these techniques. The most important step in determining if newly developed multi-omics techniques can accurately predict patient responses is prospective validation in interventional randomized trials. Despite the long path of multi-omics approaches to clinical use, we should retain expectations in this field of research.

## References

[B1] Akhoundova D. Rubin M. A. (2022). Clinical application of advanced multi-omics tumor profiling: shaping precision oncology of the future. Cancer Cell. 40 (9), 920–938. Epub 20220901. 10.1016/j.ccell.2022.08.011 36055231

[B2] Baysoy A. Bai Z. Satija R. Fan R. (2023). The technological landscape and applications of single-cell multi-omics. Nat. Rev. Mol. Cell. Biol., 1–19. Epub 2023/06/07. 10.1038/s41580-023-00615-w PMC10242609 37280296

[B3] Chai H. Zhou X. Zhang Z. Rao J. Zhao H. Yang Y. (2021). Integrating multi-omics data through deep learning for accurate cancer prognosis prediction. Comput. Biol. Med. 134, 104481. Epub 20210509. 10.1016/j.compbiomed.2021.104481 33989895

[B4] De Nola R. Loizzi V. Cicinelli E. Cormio G. (2021). Dynamic crosstalk within the tumor microenvironment of uterine cervical carcinoma: baseline network, iatrogenic alterations, and translational implications. Crit. Rev. Oncol. Hematol. 162, 103343. Epub 2021/05/01. 10.1016/j.critrevonc.2021.103343 33930531

[B5] Dentro S. C. Leshchiner I. Haase K. Tarabichi M. Wintersinger J. Deshwar A. G. (2021). Characterizing genetic intra-tumor heterogeneity across 2,658 human cancer genomes. Cell. 184 (8), 2239–2254.e39. Epub 2021/04/09. 10.1016/j.cell.2021.03.009 33831375 PMC8054914

[B6] Duan Q. Zhang H. Zheng J. Zhang L. (2020). Turning cold into hot: firing up the tumor microenvironment. Trends Cancer 6 (7), 605–618. Epub 20200321. 10.1016/j.trecan.2020.02.022 32610070

[B7] Hasin Y. Seldin M. Lusis A. (2017). Multi-omics approaches to disease. Genome Biol. 18 (1), 83. Epub 20170505. 10.1186/s13059-017-1215-1 28476144 PMC5418815

[B8] Hsieh W. C. Budiarto B. R. Wang Y. F. Lin C. Y. Gwo M. C. So D. K. (2022). Spatial multi-omics analyses of the tumor immune microenvironment. J. Biomed. Sci. 29 (1), 96. Epub 20221114. 10.1186/s12929-022-00879-y 36376874 PMC9661775

[B9] Jiang P. Sinha S. Aldape K. Hannenhalli S. Sahinalp C. Ruppin E. (2022). Big data in basic and translational cancer research. Nat. Rev. Cancer 22 (11), 625–639. Epub 2022/09/06. 10.1038/s41568-022-00502-0 36064595 PMC9443637

[B10] Lei X. Lei Y. Li J. K. Du W. X. Li R. G. Yang J. (2020). Immune cells within the tumor microenvironment: biological functions and roles in cancer immunotherapy. Cancer Lett. 470, 126–133. Epub 20191112. 10.1016/j.canlet.2019.11.009 31730903

[B11] Liu J. Lichtenberg T. Hoadley K. A. Poisson L. M. Lazar A. J. Cherniack A. D. (2018). An integrated tcga pan-cancer clinical data resource to drive high-quality survival outcome analytics. Cell. 173 (2), 400–416.e11. Epub 2018/04/07. 10.1016/j.cell.2018.02.052 29625055 PMC6066282

[B12] Liu J. Wang Y. Mei J. Nie S. Zhang Y. (2021). Identification of a novel immune landscape signature for predicting prognosis and response of endometrial carcinoma to immunotherapy and chemotherapy. Front. Cell. Dev. Biol. 9, 671736. Epub 2021/08/10. 10.3389/fcell.2021.671736 34368124 PMC8343236

[B13] Liu J. Chen C. Geng R. Shao F. Yang S. Zhong Z. (2022). Pyroptosis-related gene expression patterns and corresponding tumor microenvironment infiltration characterization in ovarian cancer. Comput. Struct. Biotechnol. J. 20, 5440–5452. Epub 20220928. 10.1016/j.csbj.2022.09.037 36249562 PMC9535418

[B14] Mao W. Cai Y. Chen D. Jiang G. Xu Y. Chen R. (2022). Statin shapes inflamed tumor microenvironment and enhances immune checkpoint blockade in non-small cell lung cancer. JCI Insight 7, e161940. Epub 2022/08/10. 10.1172/jci.insight.161940 35943796 PMC9675559

[B15] Mei J. Cai Y. Wang H. Xu R. Zhou J. Lu J. (2022). Formin protein Diaph1 positively regulates Pd-L1 expression and predicts the therapeutic response to anti-Pd-1/Pd-L1 immunotherapy. Clin. Immunol. 246, 109204. 10.1016/j.clim.2022.109204 36503156

[B16] Mei J. Fu Z. Cai Y. Song C. Zhou J. Zhu Y. (2023). Sectm1 is upregulated in immuno-hot tumors and predicts immunotherapeutic efficacy in multiple cancers. iScience 26, 106027. 10.1016/j.isci.2023.106027 36818292 PMC9932126

[B17] Murai H. Kodama T. Maesaka K. Tange S. Motooka D. Suzuki Y. (2023). Multiomics identifies the link between intratumor steatosis and the exhausted tumor immune microenvironment in hepatocellular carcinoma. Hepatology 77 (1), 77–91. Epub 20220617. 10.1002/hep.32573 35567547 PMC9970024

[B18] Sanchez-Vega F. Mina M. Armenia J. Chatila W. K. Luna A. La K. C. (2018). Oncogenic signaling pathways in the cancer genome Atlas. Cell. 173 (2), 321–337.e10. Epub 2018/04/07. 10.1016/j.cell.2018.03.035 29625050 PMC6070353

[B19] Tiwari A. Trivedi R. Lin S. Y. (2022). Tumor microenvironment: barrier or opportunity towards effective cancer therapy. J. Biomed. Sci. 29 (1), 83. Epub 20221017. 10.1186/s12929-022-00866-3 36253762 PMC9575280

[B20] Wang R. Xiong K. Wang Z. Wu D. Hu B. Ruan J. (2023). Immunodiagnosis - the promise of personalized immunotherapy. Front. Immunol. 14, 1216901. Epub 2023/07/31. 10.3389/fimmu.2023.1216901 37520576 PMC10372420

